# Progress in serology and molecular biology of equine parasite diagnosis: sustainable control strategies

**DOI:** 10.3389/fvets.2025.1663577

**Published:** 2025-09-04

**Authors:** Tengyu Wang, Xindi Chen, Xu Yan, Ya Su, Wa Gao, Chunxia Liu, Wenlong Wang

**Affiliations:** ^1^Key Laboratory of Animal Disease Clinical Diagnosis and Treatment Technology, College of Veterinary Medicine, Inner Mongolia Agricultural University, Hohhot, China; ^2^National Key Laboratory of Agricultural Microbiology, Huazhong Agricultural University, Wuhan, China; ^3^Department of Husbandry and Veterinary, Ulanqab Vocational College, Ulanqab, China; ^4^Inner Mongolia Key Laboratory of Tick-Borne Zoonotic Infectious Disease, Department of Medicine, Hetao College, Bavan Nur, China; ^5^Key Laboratory of Animal Disease Clinical Diagnosis and Treatment Technology, College of Life Science, Inner Mongolia Agricultural University, Hohhot, China

**Keywords:** horses, parasites, serology, molecular biology, diagnosis, sustainable control

## Abstract

Internal parasitic infections are a persistent challenge for horse owners, in the absence of effective vaccines and the growing challenge of drug resistance, leading many researchers to view current control strategies as unsustainable. Despite slow progress over the past two decades, effective parasitic diagnosis remains crucial for controlling infections and preventing the growing issue of drug resistance. This review examines the research progress in serological and molecular biological diagnostic methods for major equine parasites. Currently, most diagnostic techniques are based on genes such as ITS1, ITS2, COI, and IGS, which have been applied to equine strongylids, including *Strongylus* spp., *Cylicocyclus* spp., and *Cylicostephanus* spp. These methods are particularly suitable for large-scale epidemiological studies and rapid species identification. Although many diagnostic methods have been developed, most remain confined to laboratory research and have seldom been used for real-time field diagnostics. Future research should prioritize precise diagnostic methods and clinically applicable alternatives. Additionally, whole genome sequencing has been widely used in eukaryotes for population genetics and the development of diagnostic markers. However, comprehensive genomic data on parasitic species infecting equines is still limited. With the decrease in sequencing costs in the post-genomic era, a growing number of genome assemblies are expected to be released soon. These genome maps will offer comprehensive genomic data to identify specific genetic markers and variations associated with parasitic infections, enabling more accurate and reliable diagnostic techniques. High-throughput sequencing technologies will significantly accelerate progress in equine parasitology research and the development of diagnostic tools like enzyme-linked immunosorbent assay (ELISA) and TaqMan quantitative PCR (qPCR). At the same time, this paper also provides some insights into the research direction of sustainable control programs and equine parasite diagnostic methods.

## 1 Introduction

It is estimated that approximately 116 million equines (donkeys, horses, and mules) are distributed globally. According to FAO data, the number of equines has remained relatively stable over the past 50 years (1961–2019). World Mapper estimates that the global horse population will reach about 60 million by 2024. Despite millions of years of biological evolution, Equus remains highly susceptible to multiple internal parasites due to its unique living habits and feeding conditions. More than 60 common parasites, including protozoa, trematodes, cestodes, and nematodes, are known to cause parasitic infections (1). In most cases, equines exhibit symptoms similar to those of other livestock when infected with internal parasites, such as abdominal swelling, nutritional deficiencies, and weight loss. These symptoms can be assessed through body condition scoring, and are often accompanied by digestive disorders, poor appetite, and stunted growth, especially in immunocompromised foals (2). During gastrointestinal parasitic infections, equines may experience disruptions in their intestinal microbiota and impaired nutrient absorption, in addition to clinical signs. This results in increased feed conversion ratios and a higher demand for proteins and amino acids. Incorrect feeding of concentrates and changes in diet formulation may also result in the occurrence of “Friday disease” (3, 4).

For a long time, parasitic detection methods have traditionally relied on microscopic examination and necropsy of collected parasite tissues (5, 6), fecal samples (7, 8), and blood smears (9–12), thus enabling the diagnosis of internal and external parasitic infections in livestock. Some equine parasites, such as *Parascaris equorum* (*P. equorum*), *Anoplocephala perfoliata* (*A. perfoliate*), and *Oxyuris equi*, expel their eggs with feces, while others, such as the horse stomach bot fly, are expelled only when the larvae mature and the infection load becomes substantial. Furthermore, parasitic examinations of horses require trained personnel to collect feces and perform microscopic examination, necessitating the individual restraint and examination of each horse, which makes it impractical for analyzing large populations. Due to the lack of simple and effective internal parasite detection methods, deworming in equines is typically performed 2–4 times per year, depending on their use. Since the discovery of anthelmintics, animal husbandry has largely relied on the frequent use of chemical drugs, such as Benzimidazoles (BZDs), macrolides (MLs), and tetrahydropyrimidines, for year-round parasitic control (13, 14). Most competitive sport horses undergo deworming four or more times a year to enhance performance, which has contributed to the growing issue of resistance to deworming drugs. Anthelmintic Resistance (AR) refers to the inability of previously effective drugs to kill parasite populations when exposed to therapeutic doses (15). Over time, sensitive parasites transmit drug resistance to their offspring through genetic mechanisms, leading to the loss of sensitivity in previously susceptible populations (16). As the livestock industry has rapidly developed, the prevalence of drug resistance has become particularly severe, especially in cattle (17, 18), sheep, and goats (19, 20), which are economic animals. In recent years, an increasing number of clinical and epidemiological reports have described the occurrence of AR in various parasites of equines (21–23). Global equine parasitic epidemiological surveys report resistance to these four classes of anthelmintics/dewormers in over 70 species of parasites, including nematodes, *Gasterophilus* spp., and *P. equorum* (24–26), further highlighting the need to reassess parasite control strategies.

While there are differing opinions on the mechanisms behind the development of drug resistance, most researchers agree on the importance of early monitoring of parasitic infections. Early detection and control of parasitic infections and AR are critical. Many proposed deworming programs recommend regular population-wide testing using fecal samples; however, this poses a challenge for equine-specific parasites that are not detectable through fecal egg analysis. The rapid evolution of AR necessitates the urgent development of efficient and standardized diagnostic methods for internal parasites (27). Early and accurate identification of parasitic infections in individuals, along with an understanding of their development mechanisms, will help implement sustained and effective deworming programs and may assist in slowing the development of resistance. Additionally, the development of *in vitro* diagnostic methods is mostly based on the development of host-parasite immune-related protective antigens, which will also contribute to the development of new anthelmintic drugs and vaccines. In the foreseeable future, parasites in the body will still need to be controlled using chemical drugs for a long time. Although many researchers are exploring some alternative strategies to combat parasitic infections, such as breeding resistant varieties, increasing protein supplementation, and implementing “low-parasite” pasture strategies, most methods to counter resistance focus on boosting the host's immunity to enhance parasite resistance. Research has shown that adding certain bioactive substances to the diet can play a significant role in reducing parasitic infections and preventing AR development (28). However, while this approach can mitigate some of the impact parasites have on livestock traits, it may also lead to increased feed consumption. Currently, many *in vitro* diagnostic methods are used in parasitic research on ruminants such as cattle and sheep, and these methods can also detect host AR. However, related studies have few examples of parasites in horses, such as *P. equorum, Strongylus vulgaris* (*S. vulgaris*) and *Gastrophilus* spp., which can also allow us to obtain a lot of reference from the study of ruminants in the diagnostic test of horse parasites and the development mechanism of AR. However, it is worth thinking that the delay of related research also further illustrates that the development of related detection methods, with many areas in need of improvement. The purpose of this review is to systematically describe the diagnostic methods of common parasites in horses, focusing on *in vitro* detection techniques based on serology and molecular biology, and to explain their application and scientific strategies in diagnosing horse parasites, aiming to support the prevention and control of AR in horses. Additionally, this review presents opinions on the development of the horse industry and the sustainable control of parasites in equine animals in the future.

## 2 Serological detection

### 2.1 Overview of serological detection and its role in the diagnosis

Serology mainly achieves the purpose of detection and diagnosis by detecting antibodies in serum, plasma, saliva, semen, or cerebrospinal fluid of the host (29, 30). In some clinical settings, serological testing remains the gold standard for diagnosing parasitic infections when biological samples cannot be directly obtained. Although serological analysis is similar to microscopic examination in terms of operational convenience and test turnaround time, for parasites such as *Babesia* and *Theileria*, which are difficult to accurately identify on blood smears, or some horses infected with *Trypanosoma cruzi*, these individuals may be mild or asymptomatic, serological analysis can show higher sensitivity and specificity, which is crucial for the diagnostic process (31). The parasitic stage in the host triggers both nonspecific and specific immune responses, which are influenced by the location of the parasite within the host, the host's health condition, and the characteristics of the parasite antigens. Therefore, a deep understanding of the host's immune response is critical in the development of serological diagnostic methods (32). Equine parasites are typically obligate parasites that remain in the host for extended periods. Their life cycles primarily occur within the host, with eggs or adult parasites expelled in the feces. Among the current diagnostic methods, serological diagnostics serve as an effective alternative to parasitological examinations and autopsy diagnoses. During the larvae are in the migration stage in the host or fail to find signs of parasitic infection on the body surface, serological detection is a simple and effective method for rapid diagnosis of parasitic infection. As large animals, horses typically do not experience significant pain or noticeable damage to the host's body from internal parasitic infections, making it difficult for horse owners to determine whether their horses are infected with parasites through daily observation. However, during the early stages of parasitic infection (especially during the larval development stage), the establishment of early diagnostic methods could provide strong support for timely drug treatment and intervention, preventing or mitigating the development of resistance.

### 2.2 Host immune mechanisms in parasitic infections

At present, studies on ruminants infected with liver flukes have shown that during the parasitic infection of the host, both humoral and cell-mediated immune responses occur, and there is a complex interaction between the parasite and the host's immune system after infection (33). Common equine parasitic nematodes such as *P. equorum, Strongyloides westeri*, and *Gastrophilus* spp. regulate the immune system by releasing soluble mediators. These mediators interact with the host's immune cells and molecules, leading to the expression of secretory proteins and carbohydrates. In nematode studies, it has been clarified that cysteine and serine protease inhibitors (cystatins and serpins) can block T-cell responses, regulate macrophage function, and alter the activity of eosinophils and neutrophils (34). The regulation of the host immune system is both rapid and sustained, allowing parasites to persist for extended periods within the host. During the process of maintaining immune system homeostasis, both parasites and host-derived damage-associated molecular patterns are crucial for protective immune responses and tissue repair mechanisms. In studies involving fly larvae parasites, the life cycle and parasitic stages of *Hypoderma bovis* (*H. bovis*) share many similarities with certain obligate equine parasites, such as the horse stomach bot fly. The larvae of *H. bovis* can penetrate the host's skin after hatching, triggering an immune response. Currently, immunological techniques detecting excretory-secretory antigens (ESA) specific to parasites can identify anti-parasitic antibodies within 3–5 weeks post-infection (35, 36). Although natural ESA are immunogenic, their accuracy and sensitivity are limited, especially in low-intensity infections, where they are particularly ineffective, especially in large animals like cattle and horses, where such methods provide only coarse diagnostic results. In contrast, recombinant antigens exhibit greater sensitivity and specificity than natural crude antigens. Their standardized production not only improves yield but also has significant cost-control implications (37). However, despite its diagnostic value, serological testing faces limitations in large-scale epidemiological surveys. For instance, after horses undergo systematic deworming treatment, antibody titers remain elevated for an extended period, which can significantly affect serological test results and lead to a considerable number of “false positives” (38).

### 2.3 Limitations and challenges of current serological methods

Currently developed serological diagnostic methods include the complement fixation test (CFT), agglutination test (AT), indirect immunofluorescence assay (IFA), Western blot, and enzyme-linked immunosorbent assay (ELISA). These methods significantly enhance diagnostic sensitivity. A highly sensitive and specific ELISA method for detecting *Fasciola hepatica* has been developed, based on recombinant *F. hepatica*-specific antigens (39). These antigens include enzymes essential for the parasite's survival within the host, such as glutathione S-transferases (40–42). Research on has shown that after initial infection with *H. bovis* larvae, first-stage larvae (L1) produce trypsin and chymotrypsin. These proteases regulate the immune response by inhibiting both specific and nonspecific host reactions (43, 44). IgG antibodies in cattle typically appear around 45 days after *H. bovis* infection, peaking at approximately 18 weeks. This is critical for implementing early serological diagnostic strategies. Although the larvae emerge from the host's back skin during the third larval stage (L3), antibodies remain at a high level for about 4 months. Diagnosing during this period is essential for formulating an effective, scientifically informed deworming strategy (45). However, the prevalence of parasitic diseases is often associated with poor pasture management or prolonged exposure to parasitic-contaminated environments. Most horses on pastures or farms are wild and free-roaming, which presents significant challenges for tasks such as restraining horses and collecting blood samples. The collection process may result in varying degrees of injury to the horses. Additionally, these diagnostic methods also have inherent limitations, such as cross-reactivity, which can lead to false-positive results.

## 3 Molecular biology detection

### 3.1 Limitations of traditional diagnostic methods

Since the advent of the optical microscope, microscopic examination has been considered the most commonly used and economical gold standard for clinical parasitic diagnosis. Parasitological research originated from taxonomic studies, where researchers identified species by examining the morphological characteristics of parasites under the microscope. Although simple and cost-effective, this method has several limitations. For instance, it is labor-intensive, time-consuming, requires highly trained personnel, and makes it difficult to distinguish subtle differences between closely related species (46), During the microscopic examination of roundworms and nematodes, FEC values do not always correlate with worm burden (47), leading parasitologists to increasingly explore molecular biology techniques, such as gene amplification. As previously mentioned, immunodiagnostic tests have significant limitations. Currently, there are no commercially available or FDA-approved antibody tests for diagnosing parasitic diseases, such as cryptosporidiosis, schistosomiasis, and African trypanosomiasis. Commonly used antigen preparations include crude proteins, recombinant purified proteins, adult parasites, eggs, and non-standardized test antigens, leading to significant variability in experimental outcomes. In the development of diagnostic methods for *Theileria haneyi* (*T. haneyi*), since *T. equi* and *T. haneyi* are closely related, the current equi merozoite antigen 1 -based competitive ELISA used in the U.S. detects *T. equi* but not *T. haneyi*. Previous studies on serological diagnostic methods have found that immunoblot results show antigen cross-reactivity between species within the same genus, especially in regions where more than one parasite is prevalent, leading to no results or false-positive results (48). Furthermore, when the number of parasites in the host is low, serological tests tend to have lower sensitivity, which poses significant challenges for detection. Studies have shown that circulating antigens may persist in the host for some time after systematic deworming treatment, leading to false-positive results when compared with parasitic test outcomes. Therefore, the most critical time to apply this diagnostic method is during the acute phase of parasitic infection, ensuring the accuracy and reliability of the test results (49–51).

### 3.2 Emerging molecular techniques for parasitic diagnosis

With the ongoing development of molecular biology techniques, polymerase chain reaction (PCR) technology has been increasingly applied to parasite detection. In addition to traditional PCR, multiplex PCR and nested PCR techniques have also emerged. Multiplex PCR can detect multiple sequences in a single reaction, enabling the simultaneous detection of various parasites (52). Several molecular biology-based diagnostic methods, including amplified fragment length polymorphism, restriction fragment length polymorphism (RFLP), and real-time quantitative PCR (qPCR), have been utilized in parasitic detection. Loop-mediated Isothermal Amplification (LAMP) and Luminex Multi-Analyte Profiling (xMAP) technologies are emerging as novel methods for parasitic disease diagnosis. LAMP is a rapid, isothermal DNA amplification method that operates at a constant temperature (60–65 °C) using Bst DNA polymerase. It forms stem-loop structures containing repeated target DNA sequences, enabling the amplification of DNA to high copy numbers within 1 h. Nucleic acid-based molecular techniques provide superior sensitivity and specificity compared to traditional diagnostic tests. In microscopic parasitology, species identification based solely on egg observation is challenging, especially for certain parasite intermediate hosts. Equine fasciolosis is a significant re-emerging parasitic disease of horses, donkeys, and mules, caused by trematodes of the *Fasciola* genus, infecting animals through the ingestion of encysted metacercariae found on plants. To date, no diagnostic method is considered the gold standard for detecting equine fasciolosis (53). Molecular biology techniques can detect parasitic infections sensitively from small host samples, such as blood and saliva, even in asymptomatic hosts, making them highly valuable for early diagnosis and the detection of subclinical infections (54). qPCR uses fluorescently labeled probes or double-stranded DNA-specific fluorescent dyes to analyze the fluorescence signal of amplification products in real time, eliminating the need for complex electrophoresis steps (55). Due to its rapid speed, high specificity, excellent sensitivity, broad dynamic range, and low risk of cross-contamination, qPCR is widely used in the development of diagnostic methods for various diseases. It is particularly favored by researchers (56, 57). Additionally, RT-qPCR can be used to study parasite vector capability, host infectivity, diagnostics, and drug efficacy (22). TaqMan probe-based qPCR utilizes a non-extendable DNA probe that hybridizes to a specific region within the amplicon, ensuring high specificity and protecting quantitative precision by preventing the amplification of nonspecific molecules (58). This method enables accurate assessment of infection intensity by the same parasite in different hosts, with exceptional specificity and quantitative capability. This characteristic makes it an ideal tool for assessing host infection loads, providing a scientific basis for developing individualized deworming treatment plans. Molecular biology-based diagnostic techniques allow precise quantification of infection levels, providing important references for developing more effective and accurate deworming treatment plans, thereby playing a significant role in improving treatment efficacy and reducing drug misuse.

## 4 Application of serological techniques in parasite diagnosis of equids

### 4.1 Importance of serological techniques in field diagnosis

For most equine parasites, the humoral immune response typically precedes clinical symptoms during the initial infection. Horses often display notable behavioral changes only when they experience abdominal pain or more severe limb and hoof disorders. Parasitic infections are challenging to diagnose through routine visual inspection. During microscopic examination, parasites such as *F. hepatica, Clonorchis sinensis*, and *Strongyloides* can be detected in fecal samples. However, due to intermittent shedding or sampling limitations, eggs per gram (EPG) are typically very low when using the modified McMaster method (56). A retrospective study found that horses with EPG values below 500 had significantly fewer parasites in their intestines than those suggested by the fecal egg count (FEC). In summary, fecal egg tests have issues with missed diagnoses and instability. Serological methods may exhibit cross-reactivity between phylogenetically closely related species, and combining immunochroma-tographic tests with rapid immunoenzymatic assays (RIA) can greatly enhance the specificity and sensitivity of these tests, improving diagnostic accuracy (59, 60). Most serological methods for detecting equine parasites rely on ELISA (61).

### 4.2 Serological diagnosis of *Cyathostomins*

*Cyathostomins* (small strongyles) are the most prevalent and significant parasites affecting horses (62). Over the past two decades, the Jacqueline B. Matthews team at the Moredun Research Institute in the UK has made significant progress in developing *in vitro* diagnostic methods for *Cyathostomins*. Early studies identified two diagnostic antigens (20 and 25 kDa) and developed an ELISA method based on serum-specific IgG(T) responses (63–65). Although this method exhibits low cross-reactivity with *S. edentatus and S. vulgaris*, it remains valuable as a diagnostic antigen. Subsequent studies involved constructing a cDNA library from mixed Cyathostomins species and immunoscreening, resulting in the identification of the gut-related larval antigen Cy-GALA protein, which acts as an immune marker for the developmental larval stage. This antigen is exclusively expressed during the larval stage and is the first diagnostic method capable of detecting the latent phase of *Cyathostomins* (66). Further research focused on recombinantly expressing two proteins from 14 *Cyathostomins* species, leading to the selection of an antigen cocktail (Cocktail 3; CT3), which, based on specific recombinant antigen IgG(T), provides a standard curve to generate a “serum score” for each horse. The results demonstrated excellent detection performance (receiver operating characteristic area under the curve values >0.9), making it suitable for diagnosing horses infected with over 1,000–10,000 *Cyathostomins* in the mucosa and intestines, with sensitivity and specificity greater than 90 and 70%, respectively, making it commercially viable (67).

### 4.3 Serological diagnosis of *A. perfoliate*

Early studies on *A. perfoliate* involved serological research on scolex antigens (68), whole worm extracts, and excretory-secretory (ES) antigens (69). However, crude protein antigens, although easy to prepare, complicate the protein composition, with at least 14 different proteins identified, predominantly low molecular weight proteins in the 31–45 kDa range. Among the crude antigen mixture, two highly immunogenic proteins (12 and 13 kDa) were identified. Researchers measured the negative threshold values for anti-12/13 kDa IgG and IgG(T) ELISA systems, with critical OD values of 0.123 and 0.103, and sensitivity values of 56 and 62%, respectively. This suggests that anti-12/13 kDa IgG(T) may more accurately predict infection intensity in *A. perfoliate* (70). However, this method also yielded negative results in horses with low infection intensity, suggesting that diagnostic factors in parasitic diseases are not solely based on the parasite presence (especially in large animals) but also depend on the host's infection intensity (71). Among common equine parasitic infections, *Anoplocephala* spp. exhibit mixed infections, with *A. perfoliata* having significantly higher infection levels than other species. Immunological studies have yet to resolve antigen cross-reactivity between *A. perfoliata* and *A. magna* (72). No cross-reactivity has been observed between 12/13 kDa ES antigens and other parasite antigens such as *A. mamillana* and *Gastrophilus* spp. (70). Currently, commercial diagnostic kits for equine tapeworm, including the Tapeworm Blood Test and Saliva Test launched in 2014 by Austin Davis Biologics Ltd., are based on these 12/13 kDa ES antigens. The saliva test has a sensitivity of 83% and specificity of 85%, with no misdiagnoses when detecting infections with more than 20 adult tapeworms, yielding results comparable to serological tests (73).

### 4.4 Serological diagnosis of *Gastrophilus*

Jin et al. developed an indirect ELISA detection method using crude L3 antigens from *Gastrophilus pecorum* and salivary gland antigens. Only salivary gland antigens could be used for fecal sample detection, exhibiting high specificity (100%) and sensitivity (83.33%), while crude protein may non-specifically bind with other substances in the feces, hindering direct antigen detection (74).

### 4.5 Serological diagnosis of *P. equorum*

*P. equorum*, a nematode that threatens foal health, begins shedding eggs approximately 10–15 weeks after the initial infection (75). Serological diagnostic tools are crucial for assessing the likelihood of latent *P. equorum* infections. Currently, many parasite ES products from *Ascaris* species are utilized in antigen-based diagnostic development (76), including those from *Ascaris. Lumbricoides* (77) and *Ascaris suum* (78). In previous studies on *P. equorum* ES products, the fifth stage (L5) of *P. equorum* has a molecular weight range of 12–189 kDa, while L2/L3 products range from 12 to 94 kDa. Blood samples from two naturally infected foals showed antigen recognition at approximately 19, 22, 26, and 34 kDa for ES products in Silver-stained SDS-PAGE analysis (79). However, IgG(T) antibodies were not detected in foal serum prior to colostrum ingestion.

### 4.6 Challenges and considerations in serological testing

According to the performance assessment by Gasser et al. (80), ES antigens, when used as the capture layer in ES-ELISA, show significantly higher sensitivity than the other two antigens, although this increased sensitivity compromises antigen specificity. Acquiring a detailed treatment history is especially important for serological testing. Common deworming drugs, such as MLs, do not provide prolonged deworming effects, and IgG levels typically decrease over a period of 6 months following treatment (81, 82). It is generally recommended to conduct serological testing at least 4 months after treatment. A key issue in serology is the potential for antibody cross-reactivity. Although current serological diagnostic methods have achieved acceptable specificity, identifying and accurately expressing target antigens, as well as ensuring their high quality, are critical for their diagnostic application (83). We hope that equine parasite serology will advance to the same level of advancement as virology and bacteriology. Table 1 summarizes the advantages of various serological detection methods in parasite diagnosis and the main challenges faced by the current technology. Additionally, the current research progress and factors influencing future developments are summarized.

**Table 1:** Comparison of different serological methods.

**Serologic method**	**Core principle**	**Application fields and technical advantages**	**Current challenges**	**Future development direction and potential**	**Application example**
ELISA	Based on antigen-antibody reaction, parasite-associated antibodies were detected by enzyme labeling reaction	The operation is simple, the detection sample requirements are low, the low concentration detection is suitable for large-scale sample detection	It is not sensitive to acute infection. It is difficult to detect when the antibody concentration is low. The preparation cost of monoclonal antibody is high, and it needs to be detected immediately	Improve sensitivity and develop efficient detection reagents for low antibody titers	(39, 48, 63, 67, 73, 187–189)
RIA	Radioimmunoassay was used to detect antigen by radiolabeled antibody	High specificity and sensitivity, suitable for quantitative analysis	The use of radioactive materials has safety risks and complicated operation	Explore non-radioactive alternative technologies to simplify operations and expand applications	(190–192)
IFAT	Immunofluorescence antibody method was used to detect parasite antibodies	High sensitivity, suitable for accurate diagnosis	Need professional equipment, operation must be set at the same time the control, easy to other antigen cross reaction, the result may be affected by the operator	Optimize reagents and develop a simpler detection platform	(193–197)
CFT	Serological test, diagnosis by detection of antibodies or antigens	High sensitivity, suitable for large-scale detection	It is not sensitive to acute infection, and the detection process and result analysis take a long time. The non-specific binding of complements can easily lead to false positive results	Combined with other immunological techniques, a comprehensive diagnostic method was developed	(198–201)

## 5 Application of molecular biology techniques in parasite diagnosis of equids

### 5.1 Traditional PCR-based molecular diagnostic methods

In recent years, significant progress has been made in parasitic research of great public health and veterinary importance, particularly in phylogenetic systematics and diagnostics, due to ongoing advancements in life sciences and technology. The application of molecular biology has introduced new approaches and methods for the early diagnosis and precise control of parasites. Studies aimed at differentiating *A. magna* and *A. perfoliata* have shown that both parasites, whether in crude or recombinant protein form, contain low molecular weight immunoreactive components in their antigens. These components are recognized by *Anoplocephala*-positive sera at the genus level, but not at the species level (84). Currently, no reliable serological assays are available to differentiate *A. magna* from *A. perfoliata*, primarily due to the absence of species-specific antigens. This diagnostic limitation highlights the critical importance of molecular-based approaches in differentiating these closely related species. Several PCR-based diagnostic methods have been developed for equine parasite detection, primarily utilizing ITS1 and ITS2 sequence regions. These two sequences, employed as phylogenetic molecular markers in molecular classification and phylogenetic diagnosis, have significantly corroborated taxonomic frameworks derived from traditional morphological and life history analyses (85). To confirm infections in equines caused by *S. vulgaris, S. equinus, A. perfoliata*, and *Parascaris* species, fecal egg counts are commonly conducted, but the identification of the parasite species causing the infection cannot be determined by egg morphology alone. Accurate diagnosis often requires culturing the collected feces for a certain period to allow development to the third larval stage (L3). DNA fingerprinting techniques, such as restriction fragment length polymorphism (RFLP), single-strand conformation polymorphism (SSCP), and random amplified polymorphic DNA (RAPD), have been widely used in the development of molecular diagnostic methods for equine parasites, due to their speed, cost-effectiveness, and the fact that they do not require complete genome information. The fundamental principle of RFLP involves the digestion of DNA fragments from target genes using specific restriction endonucleases. Genotypes are identified based on the electrophoretic patterns of the resulting digestion products, eliminating the need for further sequencing or sequence alignment analysis (86). In 1995, Campbell et al. first used PCR-RFLP technology to amplify the ITS2 region of *Strongylus* spp., and the results indicated that the ITS2 sequence, as a diagnostic target gene, exhibits low intraspecific variation and high interspecific variation. This study laid the foundation for the ITS2 region as the basis for molecular identification. Additionally, RFLP technology in this study also provided a means of separating amplified products using agarose gel based on fragment size, allowing for rapid identification of *Strongylus* spp. without sequencing or sequence analysis (87). In subsequent studies, Gasser and Monti optimized PCR-RFLP and PCR-SSCP methods to distinguish 16 species of equine strongyles (88, 89). PCR-RAPD technology has been widely used in the classification of numerous parasites (90, 91). In equine parasitic detection, this technology has been applied to define species of two *Cylicocyclus* species, such as *C. insigne* and *C. elongatus*, which are morphologically very similar but can be accurately distinguished, further demonstrating that this method is suitable for closely related species. Over the past 30 years, researchers have primarily developed diagnostic methods for equine parasites based on ribosomal RNA genes, particularly the ITS and IGS (Intergenic Spacer) regions. In studies of equine strongylids, by comparing the performance of two molecular barcodes (COI gene and ITS-2 rDNA gene) in different biological type samples, it was found that although the results of the two barcodes were fairly consistent, the PCR amplification efficiency of the COI gene was lower than that of ITS2, particularly in certain strongyle species (e.g., *Cylicostephanus calicatus* and *Cylicostephanus longibursatus*), where the COI barcode amplification efficiency was less than 70%. In group identification, the COI gene, due to its higher genetic diversity and larger sequence variation, can lead to PCR amplification bias, making it difficult to identify specific life cycle stages of some species and affecting the final sequencing results (92).

### 5.2 Emerging molecular diagnostic approaches

Hodgkinson et al. (93, 94) used Southern blot to validate the design of digoxigenin (DIG)-labeled probes targeting the IGS conserved region of four highly prevalent species (*C. ashworthi, C. nassatus, C. longibursatus, C. goldi*), achieving molecular identification of single eggs, L3 larvae, and L4 larvae. The research team later developed a PCR-ELISA method, which was completely consistent with the Southern blot results and could identify low-abundance species (e.g., *C. catinatum*, which accounted for only 1.7%). This method was used to assess the therapeutic efficacy in horses treated with BZDs. Compared to the RFLP probe method, it was more suitable for analyzing polymorphic fragments (95). The widely used Reverse Line Blot (RLB) detection method for diagnosing *strongyloid* species currently involves non-radioactive hybridization of PCR amplicons with different oligonucleotide probes in a single reaction, allowing for the detection and identification of more than 16 *strongyloid* species simultaneously (96–98). This method has also been widely applied to study the relationship between drug resistance and changes in *Cyathostomin* population structure, thereby exploring the potential mechanisms behind the shortened egg reappearance period after treatment (99, 100). Martins amplified the ITS2 DNA fragment of *S. vulgaris* by PCR and compared it with the results of fecal culture L3, demonstrating a lower consistency, further confirming that PCR diagnostic methods have become the most effective tool for diagnosing *S. vulgaris* (101). Nielsen developed a dual-labeled TaqMan qPCR assay specifically targeting *S. vulgaris*, which exhibited no cross-reactivity with other equine parasitic nematodes. This assay demonstrated a detection limit of 0.5 egg equivalents, providing a precise and efficient tool for diagnosing *S. vulgaris* infections. Similarly, Zhou et al. developed a novel real-time quantitative PCR assay based on the TaqMan-MGB probe, specifically for detecting *T. haneyi*, a pathogen causing equine piroplasmosis. The assay demonstrated excellent specificity and sensitivity, with a detection limit of 1 × 10^2^ copies/μl, and was validated by comparison with nested PCR. This new diagnostic method promises to enhance early detection and control of *T. haneyi infections*, complementing existing PCR techniques for *Theileria equi (T. equi), Babesia caballi (B. caballi)*. The LAMP-based diagnostic method for *T. equi* and *B. caballi* has been developed, providing rapid on-site results within 1 h (102). Fecal samples, the most accessible for on-site diagnosis, contain more potential inhibitors than blood samples, which can reduce the sensitivity and accuracy of the LAMP assay. Furthermore, fecal samples often require more rigorous pretreatment and ultrapure DNA extraction (103, 104). The advancement of microfluidic chip technology has enabled the successful application of isothermal amplification methods for detecting various veterinary parasites (105–107). Chen et al. (108) developed a microfluidic LAMP system that can identify five parasites. This method integrates sample pretreatment, biological separation, biochemical reactions, and signal analysis into microchips just a few square centimeters in size, achieving both miniaturization and automation in parasite detection (109, 110).

### 5.3 Next-generation sequencing for comprehensive parasitic diagnosis

With the ongoing development of high-throughput sequencing technology, next-generation sequencing (NGS) has become widely applied in the diagnosis and research of equine parasites. Mitchell used NGS technology to conduct qualitative and quantitative analysis of the species and egg abundance in equine fecal samples, replacing traditional larval culturing and necropsy methods. This also marks the first recorded instance of the equine “nemabiome” (111). Ghazanfar et al. combined NGS technology with the modified McMaster technique to study the prevalence and diversity of *gastrointestinal nematodes* in Australian racehorses. Compared to traditional microscopic examination or PCR methods, NGS proved more efficient and precise, identifying 23 *strongylid* species, including 18 cyathostomins and five large strongyle species. Using high-throughput data from NGS, the species abundance of various parasites in fecal samples was analyzed. The three most abundant species of small strongyles were *Cylicostephanus longibursatus* (28%), *Cylicocyclus nassatus* (23%), and *Coronocyclus coronatus* (23%), which together accounted for 95.8% of the entire parasite community. The study also revealed the diversity of nematode communities across different climatic zones and age groups. In foals and weanlings, the most common strongylid species was *Cylicostephanus longibursatus*, with relative abundances of 35.3% in foals and 34.1% in weanlings. Nematode community diversity in the Non-Seasonal Rainfall Zone and Winter Rainfall Zone was significantly higher than in the Mediterranean Rainfall Zone (112). Recently, Hamad et al. employed ITS-2 rDNA metabolic barcoding sequencing technology with the Illumina MiSeq platform to study the strongylid nematode populations on a large equine farm in Thailand. By analyzing fecal samples from 57 horses on the farm, they successfully identified 14 *strongylid* species, including *Cylicocyclus nassatus* and *Cylicostephanus longibursatus*. The study revealed the complexity of the strongylid nematode community and the relative abundance of species, while also validated the quantitative potential of high-throughput technology in equine parasite detection. It provides an important molecular data foundation for future drug resistance monitoring and parasite management (113, 114). Table 2 summarizes the advantages of widely used molecular biological detection methods in parasite diagnosis, along with the main challenges faced by current technologies. It also outlines the current research progress and factors influencing future developments.

**Table 2:** Comparison of different molecular biology methods.

**Molecular biology methods**	**Core principle**	**Application fields and technical advantages**	**Current challenges**	**Future development direction and potential**	**Application example**
PCR	Using DNA amplification technology to detect the genetic material of parasites	High sensitivity and specificity, can detect low concentrations of parasitic infections	Sample processing requirements are strict, only qualitative, high equipment requirements, may be affected by pollution	Develop portable PCR equipment to simplify operations and reduce on-site requirements	(52, 202–204)
Real-time PCR	Real-time quantification of DNA in the amplification process	Accurate detection, real-time quantification, suitable for viral load assessment	High equipment requirements, complex operation, sensitivity is limited by the quality of the sample	High-throughput detection, combined with portable devices, improves real-time detection capabilities	(163, 205–207)
LAMP	Amplification of specific DNA fragments by cyclic reaction	Rapid reaction, low equipment requirements, suitable for on-site rapid detection, real-time evaluation results by naked eye	Sensitive to sample contamination, there is a cross-reaction, may affect the accuracy	Combined with microfluidic chip, the development of multiple target detection platform	(208–211)
Microarray	Detection of multiple target genes by microarray	It can detect the expression of multiple parasite genes at the same time, which is suitable for comprehensive analysis and drug resistance detection of complex samples	The data processing is complex, and only samples with existing sequencing information can be detected, with high cost and low sensitivity	Improve chip sensitivity and throughput, reduce costs, and develop convenient application tools	(212–214)
RFLP	Specific DNA fragments are analyzed by restriction enzyme digestion	It can be used to study the typing and genetic diversity of parasite species	The operation is cumbersome, the amount of DNA template is high, the time is long, and the cost is high	Combined with high-throughput technology, accelerate data collection and analysis, and improve application flexibility	(87, 88, 215–219)
RAPD	By using random primers to amplify specific DNA fragments, genetic polymorphisms can be detected	The technology is simple, low cost, and does not require complete genomic information, which is suitable for rapid screening of multiple samples	The sensitivity is unstable, mainly affected by primers, and cannot provide accurate genomic location information	Combined with high-throughput technology, the accuracy of primer design is improved, which is suitable for gene diversity research	(90, 91, 220)
RLB	By using specific probes to hybridize with the target DNA, the DNA of multiple parasites was detected	It can detect multiple parasites at the same time with high sensitivity and is suitable for clinical samples	High-quality probes and samples are needed, and the control of hybridization conditions is high	Combined with microfluidic technology, the operation is simplified, the sensitivity is improved, and the detection range is expanded	(96–98, 100, 221, 222)
Next-Generation Sequencing (NGS)	The parasite genome sequence was detected by large-scale parallel sequencing technology	High precision, can carry out whole genome analysis of parasites, help to analyze the development process of drug resistance, and formulate scientific prevention and control programs	Data analysis is complex, requires strong computing power, high technical requirements, high cost	Reduce costs, improve data processing efficiency, applied to a wide range of parasite diagnosis	(111, 112, 223)

## 6 Pasture management and sustainable control strategies for equine parasites

This review provides a clear summary of current serological and molecular biological methods for detecting equine parasites. Traditional fecal examination techniques remain a key component in the development of detection methods. Serological and molecular biology-based diagnostic tools are crucial in the early detection of parasitic infections in horses. However, these methods face several limitations regarding their development and practical field application. Table 3 presents a comparative analysis of the two diagnostic methods, highlighting key factors such as sensitivity, field applicability, and commercial viability. These factors are vital for the selection of appropriate clinical diagnostic tools.

**Table 3:** comparison-of-serological-methods-and-molecular-biology-methods.

**Feature**	**Serological methods**	**Molecular biology methods**
Sensitivity	Moderate sensitivity; antibody testing is less effective in the early stages but more reliable in later stages. It may fail to detect latent or low-level infections	High sensitivity; capable of detecting early, latent, and low-level infections
Specificity	Relatively high; potential cross-reactivity among closely related species	Extremely high (under strict contamination control), with minimal cross-reactivity
Detection stages	Primarily used for chronic or asymptomatic infections, with detection dependent on the onset of immune response	Applicable for detecting infections at any stage, particularly effective for acute or latent infections
Cost	Low cost; suitable for use by non-specialized personnel	High cost; requires specialized equipment, reagents, and trained personnel
Field applicability	Suitable for large-scale testing and field use, particularly in resource-limited settings. Requires minimal laboratory infrastructure, and the developed colloidal gold method is applicable for on-site rapid detection	Limited field applicability; sample processing requires a laboratory setting. The method is highly dependent on environmental conditions, with strict temperature requirements for reagent storage and transport, and specialized equipment needed for detection
Ease of operation	Simple and rapid; no technical training required, easy to implement in clinical practice	More complex; requires specialized training and dedicated equipment
Time for results	Short sample preparation and reaction time, suitable for rapid screening	Longer sample preparation and turnaround time, especially with complex protocols such as qPCR and NGS
Commercial viability	ELISA is a well-established and widely used method, while the development of colloidal gold assays has a low entry barrier, making mass production feasible. Currently, commercial test kits are available for various parasitic infections	Molecular detection methods have a high development threshold, requiring comprehensive omics data support. The production capacity of biological reagents is limited, with a high dependency on instruments and reagents. Commercial test kits for equine parasites are limited in number
Cross-reactivity	Relatively high, particularly among phylogenetically similar species, where pathogens of the same family or genus may share similar epitopes	Exhibits a low degree of species recognition, typically based on the design of specific target sequences
Sample requirements	Requires serum or other bodily fluids, which can be used directly or subjected to simple centrifugation	Requires blood, fecal, or tissue samples, typically requiring complex steps such as nucleic acid extraction
Impact of treatment	Can be affected by previous treatments (e.g., anti-parasitic drugs)	Not affected by past treatments; reflects current infection status
Source	(36, 194, 224–234)	(92, 161, 226, 229, 232, 235–240)

Gastrointestinal parasites, particularly cyathostomins, are among the most common equine parasitic infections and are prevalent in global epidemiological surveys, with over 50 species identified (115). In pastures where horses are free-ranging, the infection rate can reach up to 100%, with most horses carrying 3–4 different internal parasites. Due to the misuse of deworming drugs and unscientific medication practices, numerous failures in control measures have occurred, and the failure of recommended treatment protocols has indicated widespread resistance to deworming medications. This poses new challenges in parasite control and resistance management. Effective parasite control requires planning and management at each developmental stage. The use of broad-spectrum dewormers, such as ivermectin, should be assessed according to pasture management conditions, reducing deworming frequency and drug dosages while avoiding frequent large doses. Scientific recommendations on whether horses need deworming should be provided through *in vitro* diagnostic methods for equine parasites. Growing resistance has created new challenges for farm and ranch owners, urgently necessitating the re-evaluation of management strategies and the formulation of scientifically sound control plans. Large-scale fecal collection and analysis should be performed, with control plans based on diagnostic results. Horses can be grouped according to parasite species and number, and high- and low-risk areas should be established. High-risk areas include high-density breeding zones and young horses (2–5 years old) raised on low-quality forage, with high FEC and initial indications of AR. Low-risk areas consist of low-density breeding or individually housed horses, fed high-quality, clean pasture, and exhibiting low FEC as confirmed by parasite testing. Horses in low-risk areas only need to maintain good feeding environments and fodder hygiene. With veterinary evaluation, the frequency of fecal egg count monitoring can be reduced in these areas. Grazing management is especially critical for herds where parasite control is failing. If rotational grazing is used, the pasture should be regularly cleaned to prevent overgrazing, which can effectively control the risk of parasitic infection.

For the past half-century, the horse industry has depended on the use of chemical drugs for deworming. In recent years, an increasing amount of research shows that uncontrolled single or frequent rotations of drugs accelerate the emergence and spread of resistant populations, rendering a limited number of drugs ineffective (116). With the rapid development of the global equine and animal husbandry industries, sustainable pasture management strategies have become a key focus. In ruminant research, many natural plants or feed additives similar to probiotics have been found to combat parasites, but research in equines is relatively limited. Beg et al. administered water-soluble herbal extracts of *Nigella sativa* (Kalwanji) seeds, *Fumaria parviflora* (Burgi Shatara) leaves, and *Fumaria macrophylla* (Jantaan) leaves via nasogastric tube on Day 0 and 18, with doses of 0.05, 0.10, and 0.15 g/kg, respectively, and evaluated efficacy based on the eggs per gram of feces (EPG). The results showed that all herbal extracts significantly reduced EPG after the first treatment on Day 18, with the best results at Day 28, demonstrating that *N. sativa* has strong anthelmintic effects against *P. equorum* (117). Payne et al. conducted crude extractions from 37 native Australian plants and tested their *in vitro* effects on the development of *Cyathostomins*. Seven plants, including *Acacia baileyana, Acacia melanoxylon, Acacia podalyriifolia, Alectryon oleifolius, Duboisia hopwoodii, Eucalyptus gomphocephala*, and *Santalum spicatum*, completely inhibited the development of *Cyathostomins* larvae, with an IC50 value of just 30.9 μg/ml to achieve 50% developmental inhibition. Other tested plant extracts displayed varying degrees of anthelmintic activity *in vitro* (118). *Alectryon oleifolius* was one of the most effective plants in inhibiting larvae. The main active component in the plant is Procyanidin A2, which, after purification, completely inhibited larval development at a low concentration of 50 μg/ml, with an IC50 value of 12.6 μg/ml. Compared to commonly used anthelmintics such as ivermectin (IC50 = 0.22 ng/ml) and levamisole (IC50 = 115 ng/ml), Procyanidin A2 exhibited excellent anthelmintic properties (119). Some studies suggest that tannins and total phenols are considered the key active components in plants for combating parasites. Tannins can bind to proline, an amino acid in the nematode's cuticle, causing larval death (120–122). *Moringa oleifera* (*M. oleifera*) leaves contain abundant vitamin E and quercetin, which protect the plant from microbial damage during growth and possess strong antioxidant activity (123–125). Mbogning-Tayo et al. (126) used *M. oleifera* leaf extract for egg hatching inhibition tests on *Haemonchus contortus* eggs, showing that at concentrations of 3.75 and 5 mg/ml of *M. oleifera* extract, the egg development was inhibited by 60.3% ± 8.2% and 92.8% ± 6.2%, respectively. Although research on *M. oleifera* components is incomplete, the maximum anti-parasitic effect likely results from the coordinated action of multiple components (127). Buono et al. extracted allicin, the active component of garlic, and conducted egg hatching inhibition assay (EHA) and larval migration inhibition tests on *Cyathostominae* eggs and L3 larvae, showing inhibitory activity. Allicin also exhibited growth inhibition in *in vitro* studies of *B. caballi* and *Theleria equi*. However, in naturally infected horses, it did not exhibit significant anti-parasitic effects (128–130). This suggests that garlic's mechanism of action may not directly kill the parasites, but rather enhance resistance by modulating the immune system. Other herbs, such as *Acacia nilotica, Rumex abyssinicus*, and *Zingiber officinale*, also exhibit strong anti-parasitic effects (131).

With growing attention to the sustainable development of grassland ecosystems and livestock, biological control-based management strategies are being increasingly implemented. *Duddingtonia flagrans* (*D. flagrans*) and *Pochonia chlamydosporia* have been shown to be effective and feasible alternatives to chemical dewormers. Horses consuming these fungi can survive in the gastrointestinal tract without disrupting the existing gut microbiota (132). After excretion with feces, predatory fungi form capture networks that significantly reduce larval contamination of pastures and the environment, thus potentially affecting the survival of nematode larvae (133–135). Commercial products based on *D. flagrans* have already been launched on the market, primarily applied through feed supplementation. Following a period of research and use, these products have significantly reduced the number of parasites in pastures (132, 136–138). Research indicates that equine gastrointestinal worm infections significantly disrupt the host's intestinal microbial (139, 140). Adding probiotic dietary supplements to the diet is undoubtedly an effective way to reduce parasitic infections in horses (141).

In many pastures where horses are free-ranging, many horse owners engage in unscientific, irregular, and non-systematic deworming practices. The need for parasite testing and guided deworming should be emphasized through public education and government regulatory oversight. A comprehensive, year-round control strategy should be developed based on regional differences and parasite distribution. Regular cleaning of feces from horses and other livestock to maintain pasture hygiene has long been considered a fundamental method for controlling equine parasitic infections. However, this task is time-consuming and labor-intensive, making it difficult for many pasture owners to adopt. Despite the current available parasite control strategies, these actions should still be undertaken.

## 7 Conclusion and future directions

Over the past 30 years, numerous diagnostic kits for identifying and diagnosing equine parasites have been developed, leading to significant advancements. However, most of these methods detect the presence of infection in horses, often requiring expensive instruments and reagents. Additionally, diagnosing parasitic diseases during their latent phase is challenging, which likely explains why few findings have been widely implemented. Although current parasitic diagnostic technologies, such as ELISA and qPCR, offer highly sensitive and specific detection methods, they still face several limitations in field applications, particularly when compared to traditional fecal egg detection methods. Main obstacles include high equipment costs, the need for specialized training, and difficulties in transporting equipment and reagents to remote regions. In contrast, although FEC testing has relatively lower sensitivity, it offers significant advantages in cost, examination time, and operational convenience, making it the most widely used screening tool in resource-limited areas. Bridging the gap between laboratory-based technologies and their field applications requires the establishment of mobile diagnostic platforms that integrate serology and molecular biology (142). The widespread adoption of smartphones has led to the development of efficient smartphone-based parasitic diagnostic platforms, including those for egg identification, remote diagnosis, and colorimetric assays (143–145). Additionally, expanding veterinary training programs is crucial to ensure on-site professionals become proficient in these diagnostic techniques, thereby enhancing their effectiveness in field applications. Automated mobile diagnostic platforms can significantly reduce labor costs. Compared to manual methods for sample extraction and preparation, automated platforms use fewer reagents and exhibit smaller variability between groups (108). Point-of-care tests (POCTs) have emerged as a practical alternative and are rapidly growing in veterinary diagnostics (146). Fecal egg count reduction tests are currently considered the “gold standard” for anti-resistance detection in horses (147–149). *In vitro* tests, including egg hatching tests and larval development tests, are also commonly used as supplementary methods for anti-resistance testing (27, 150, 151). However, it is currently difficult to define the AR profiles of various parasites in horses, as each parasite may display resistance to different classes of anthelmintics, and the mechanisms of resistance associated with each drug may vary (152, 153). Attributing this resistance to specific gene SNP mutations is not always straightforward. Furthermore, many resistance mechanisms remain poorly understood (154). Research on resistance suggests that the development of parasitic resistance is likely influenced by multiple genes and various mechanisms of action (155–157). Current diagnostic methods face significant challenges in specifically identifying resistant populations, resulting in prevention and control strategies lacking specificity. The lack of related research is associated with horses being a special non-economic livestock species. The horse parasite genomic information available from WormBase ParaSite and NCBI parasite genome databases is still limited compared to ruminant parasite genomic information, further reflecting the significant gap between research teams and funding investment.

Genomic and transcriptomic data related to equine parasites are currently scarce. Whole-genome sequencing of different parasitic species, including both sensitive and drug-resistant strains, would provide deeper insights into the quantity and mechanisms of resistance genes (158–161). Constructing genetic maps of these species' genomes to identify unique genetic markers for species identification will aid in the development of early diagnostic methods. Additionally, research on veterinary helminths has confirmed that genetic markers of AR to BZDs, Levamisole (LEV), and Monepantel are identified using known SNP markers. Expanding the genomic maps of parasitic strains will enhance the diagnosis and detection of resistance (154, 162). Serological and molecular biological techniques currently allow for the study of parasite species, population genetic diversity, and mutations between strains in different regions. In the future, multiplex qPCR diagnostic methods can be developed for common or target parasite species on pastures (163–165). This method can detect infection status in horses and estimate infection quantities and ratios, thereby assessing the AR situation of specific parasites (166). LAMP has good potential for on-site testing and has been widely applied in research on bacteria, viruses, and parasites (167–170). However, parasitic detection samples are often feces or eggs, which are small and contain numerous contaminants, posing challenges for developing effective detection methods (171).

With the rapid advancement of artificial intelligence (AI), an increasing number of researchers are integrating parasitic diagnostic techniques with artificial intelligence (AI) technology to enhance detection efficiency and accuracy. Li et al. developed a low-cost, fully automated diagnostic system that integrates a portable robotic-assisted microscope with convolutional neural networks for the counting of parasite eggs in fecal samples. The system was tested on eggs of *Eimeria, Strongyles*, and *Trichuris* spp., with an error margin of fewer than one egg per McMaster counting chamber (172). Numerous machine learning (ML) and artificial neural network-based (ANN) models have been employed for the automated identification of parasite eggs (173–175) Januário et al. proposed an innovative approach that integrates genomic selection, machine learning, and image analysis to optimize resistance to gastrointestinal nematodes (GIN) in sheep (176). This method classifies sheep into resistant, resilient, and susceptible categories using machine learning techniques such as multinomial logistic regression, random forest (RF), and artificial neural networks (ANN), while evaluating susceptibility levels across different farms based on fecal egg count (FEC), packed cell volume, and Famacha© scores (177, 178). Additionally, the RF model was applied to segment ocular conjunctiva images of sheep, automatically classifying anemia levels according to Famacha© scores, thus improving the accuracy of parasite infection detection. For genetic analysis, Bayesian methods, including BayesA, BayesB, and Bayesian Lasso (BLASSO), were employed to estimate the breeding values (EBVs) of traits related to resistance (179–181). Datasets that are continuously enriched will substantially enhance diagnostic efficiency and accuracy, and AI will offer more efficient solutions for screening parasites and drug resistance (182–186). The development of these technologies facilitates large-scale parasite control strategies for equines, benefiting more key stakeholders. Additionally, specific immune antigens and drug targets can be identified for the development of vaccines and other biological products for major parasites, providing more effective means of safeguarding horse health.

In conclusion, while there are some ambiguities in the Swedish, Danish, Dutch, and ESCCAP guidelines concerning equine parasitism and farm management, and differences in deworming timing and diagnostic testing methods due to certain policy factors, there is a shared consensus on advocating monitoring-based control programs. Countries and organizations should proactively offer continuing education webinars on parasite control, providing horse owners and stable managers with up-to-date, evidence-based information to reduce confusion from outdated knowledge. There should also be a concerted effort to implement standardized parasite control strategies across the industry, promoting diagnostic-driven deworming over traditional blanket deworming. Given the current prevalence of drug resistance, not all categories of deworming agents are available in all countries, and strengthening regulation on deworming drug use is essential to preserving their efficacy. Policies should encourage the development of new deworming drugs and explore alternative control measures, especially through long-term studies on combination drug strategies. Furthermore, fostering collaboration among veterinary schools, pharmaceutical companies, and regulatory agencies will ensure that, in the coming years, parasite control guidelines become more unified and widely adopted, effectively curbing resistance development and safeguarding horse health.
